# Perceptions towards biologic and biosimilar therapy of patients with rheumatic and gastroenterological conditions

**DOI:** 10.1186/s41927-022-00309-4

**Published:** 2022-12-23

**Authors:** Thomas Khoo, Navkiran Sidhu, Franca Marine, Susan Lester, Alannah Quinlivan, Debra Rowett, Rachelle Buchbinder, Catherine L. Hill

**Affiliations:** 1 Rheumatology Unit, The Queen Elizabeth Hospital, Woodville South, SA Australia; 2 Arthritis Australia, Glebe, NSW Australia; 3 Discipline of Medicine, University of Adelaide, Adelaide, SA Australia; 4 UniSA Clinical and Health Science, Adelaide, SA Australia; 5 Drug and Therapeutics Information Service, SALHN, Adelaide, SA Australia; 6 Department of Epidemiology and Preventive Medicine, School of Public Health and Preventive Medicine, Monash University, Melbourne, VIC Australia; 7 Monash-Cabrini Department of Musculoskeletal Health and Clinical Epidemiology, Cabrini Health, Malvern, VIC Australia; 8 Department of Rheumatology, The Royal Adelaide Hospital, Adelaide, SA Australia

**Keywords:** Biological products, Biosimilar pharmaceuticals, Health knowledge, attitudes, practice, Arthritis, Inflammatory bowel diseases

## Abstract

**Background:**

Biologic and targeted synthetic disease modifying agents (b/tsDMARDs) have broadened the treatment landscape for autoimmune diseases particularly in patients refractory to conventional DMARDs. More recently, the introduction of biosimilars has reduced the price of bDMARDs, potentially improving accessibility. Though efficacy and safety have been described, patient attitudes to b/tsDMARDs are not well-understood. We aim to investigate patients’ beliefs about biologic and biosimilar therapy, and the factors influencing their perceptions.

**Methods:**

Patient consumer groups (Arthritis Australia, Crohn’s and Colitis Australia) assisted in advertising an online questionnaire for people with a self-reported diagnosis of inflammatory arthritis (IA) or inflammatory bowel disease (IBD). The questionnaire incorporated the Belief about Medicines Questionnaire (BMQ) and the single-item literacy screener (SILS). Sources and favourability of biologic/biosimilar information were analysed, using the chi-square and a non-parametric trend test for unordered and ordered categorical variables respectively, comparing respondents with IA and IBD.

**Results:**

Eight hundred and thirty eight people (686–IA, 144–IBD, 8 both) responded. 658 (79%) used b/tsDMARDs. The BMQ demonstrated high necessity belief (median 4.2) with moderate concerns (median 2.8) about biologics. 95% of respondents obtained medication information from specialists though most used multiple sources (median 4). The most positive resources were specialists and specialist nurses. 73/141 (52%) respondents with IBD obtained information from specialist nurses compared with 202/685 (29%) with IA (*p* = 0.012). Respondents with limited reading ability on SILS were more likely to discuss information with a general practitioner or pharmacist. Younger respondents and those with higher BMQ concern scores more frequently consulted less reliable sources (e.g. social media). 502 respondents (60%) answered the biosimilar questions. Only 23 (4.6%) reported currently using a biosimilar and 336 (66.9%) were unsure if biosimilars were available in Australia. Specialist recommendation was the most frequent factor that would influence a patient to change from originator to biosimilar (352/495, 71.1%).

**Conclusions:**

There is a high level of trust in specialists’ recommendations about b/tsDMARDs, although most people also utilise additional information sources. Contextual factors influencing resource selection include age, reading ability and degree of concern about medicines. People with IA and IBD have similar attitudes though those with IBD more frequently access specialist nurse advice.

**Supplementary Information:**

The online version contains supplementary material available at 10.1186/s41927-022-00309-4.

## Background

Biologic therapies are organic molecules, derived in part or whole from living organisms, which have revolutionised the treatment of autoimmune diseases. They offer potent, specific approaches which translate our knowledge of molecular mechanisms of disease to therapeutic targets [1]. Since the advent of etanercept, the first TNF-inhibitor approved for use in rheumatoid arthritis (RA), there has been a proliferation of biologics exploiting different molecular targets. Currently, fifteen biologic or targeted synthetic DMARDs (b/tsDMARDs) are available in Australia under the Pharmaceutical Benefits Scheme (PBS) for the treatment of inflammatory arthritis (IA) (abatacept, adalimumab, baricitinib, certolizumab pegol, etanercept, golimumab, guselkumab, infliximab, ixekizumab, rituximab, secukinumab, tocilizumab, tofacitinib, upadacitinib and ustekinumab) and five for the treatment of inflammatory bowel disease (IBD) (adalimumab, infliximab, tofacitinib, ustekinumab and vedolizumab).

In Australia, the national government reimburses medication costs under the PBS. For medications such as b/tsDMARDs, authorisation is required to ensure that initially, specific criteria are met demonstrating ongoing clinical and biochemical disease activity despite first line therapies, and subsequently, that the b/tsDMARD is having sufficient benefit to justify continuation. For bio-originators and the initial prescriptions of biosimilars, this involves the submission of an application form for approval every four (initial prescription) and six (subsequent prescriptions) months. For the continuation of biosimilars there is a streamlined authority code which encourages biosimilar uptake by prescribers.

The use of biologics is increasing across different specialties and organ systems. Some, such as the TNF-inhibitors, are used in both IA and IBD whereas others are specific for a single indication (for example, vedolizumab, an α_4_β_7_ integrin antagonist, for IBD alone). In both rheumatology and gastroenterology, b/tsDMARDs have profoundly changed the landscape of treatment, shifting reliance on traditional immunosuppressants and glucocorticoids, and providing a number of effective options for often debilitating autoimmune inflammatory diseases [2].

Biologics are complex molecules with proprietary manufacturing techniques, meaning that they are expensive to produce. Biosimilars are highly similar versions of already registered biological medications which have been shown to have near-identical chemical, biological, efficacy and safety characteristics as the originator drug. Although they are not identical copies of the originator, regulatory approval processes require biosimilars to demonstrate that there are no clinically meaningful differences between the biosimilar and the reference biologic [3].

The introduction of biosimilars to the Australian pharmaceutical market has reduced the cost of biologics which may lead to easier access to these medicines with fewer restrictions in future [4]. However, uncertainty regarding the concept of biosimilars and their regulatory process has created controversy around the prospect of switching from originators to biosimilars [5]. Nevertheless, the use of biosimilars is increasing in rheumatology practice in Australia. For rheumatoid arthritis, when etanercept was first included under the PBS in 2017, the biosimilar accounted for 2.8% (186/6566) of total etanercept prescriptions compared with 39% (2907/7533) in 2021. For adalimumab, which became available on the PBS in 2021, biosimilars accounted for 19.9% (2387/11995) of total adalimumab prescriptions in that year. The rituximab originator has now been removed from the PBS in April 2021 and only the biosimilar is available [6].

Global experiences of biosimilar uptake vary. In the USA, an infliximab biosimilar entered the market at the end of 2016 but had sparse uptake, accounting for only 0.9% of TNF inhibitor use at the start of 2019 compared with bio-originator infliximab at 19.6% of TNF inhibitor use [7]. On the other end of the spectrum, some countries have implemented mandatory non-medical switching, such as Denmark where uptake of biosimilar infliximab was 97% within the first year and subsequent biosimilar etanercept and adalimumab had similar uptake within six months of bio-originator patent expiration [8].

While the clinician, public health and governmental perspectives on biologics and biosimilars have previously been articulated in the literature, exploration of the patient perspective is lacking.

Patient attitudes towards biosimilars significantly influence adherence and the experience of a nocebo effect after switching from biologic originator [9] and consequently, understanding the foundations of their perceptions represents an important step towards framing messages appropriately and effectively [10]. Furthermore, though patients’ and their physicians’ attitudes have previously been compared [11], a comparison of patient groups with IA and IBD, who see different specialists but use similar biologics, has not previously been performed.

We sought to determine patients’ opinions and from where they obtained information, as well as compare the views of patients with IA and with IBD.

## Methods

### Study respondents and design

People with a self-reported diagnosis of IA (rheumatoid arthritis (RA), psoriatic arthritis (PsA), ankylosing spondylitis (AS)) or IBD (Crohn’s Disease, ulcerative colitis) were eligible for inclusion. A web link to complete a voluntary online anonymous survey (Survey Monkey, https://www.surveymonkey.com) was made available and promoted through the patient consumer groups, Arthritis Australia and Crohn’s and Colitis Australia. The surveys were open for completion over a five-week period between April 24 and June 1, 2020. Responses were excluded if patients did not identify having a diagnosis of IA or IBD, had never heard of biologics, did not respond to any of the questions or did not provide any relevant medication history.

This research was conducted according to the Australian National Statement of Ethical Conduct in Human Research (2007) incorporating all updates as well as the 1964 Declaration of Helsinki and its later amendments. Ethics approval was obtained from the Central Adelaide Local Health Network Human Research Ethics Committee, reference number 12423. All methods were carried out in accordance with relevant guidelines. Informed consent was obtained from all respondents.

### Questionnaire

The full questionnaire is available as an Additional file 1. It was designed and developed by a group of rheumatologists, a pharmacist and a representative of the patient consumer groups. It was user tested by a small group of people with IA prior to wider release.

Only respondents who self-reported a diagnosis of IA or IBD were able to proceed with the questionnaire. The questionnaire collected cross-sectional demographic and disease characteristic data and the following information in order of presentation:

1. Sources of information: respondents were asked where they obtained information about biologics. Response options included: various health care professionals (specialists, general practitioners, nurses and pharmacists); non-healthcare-related contacts (relatives/friends and other patients) and various forms of media (educational websites such as those produced by the Australian Rheumatology Association, Arthritis Australia or Crohn’s and Colitis Australia; social media (e.g. Facebook, Twitter, Instagram); chat rooms; newspapers; magazines; television and radio). For each nominated source, patients were asked to rate how favourable this resource was towards biologics on a scale of 1 (very negative) to 10 (most positive).
2. Biosimilars: respondents were asked how familiar with biosimilars they were (with five ordered options ranging from very familiar’ to ‘never heard of them before’) and whether biosimilars were available in Australia. Following these questions, a short description of biosimilars was given. This included how they are developed and their intention of providing similar effects to their biologic originators. Respondents were then asked about their opinions on changing to a biosimilar and what factors might influence this decision (with options including specialist recommendation, supportive clinical trial evidence, cost to self or government, and convenience).
3. The Beliefs about Medicines Questionnaire (BMQ) [12] applied to biologics. The BMQ comprises two sections: the BMQ-Specific which assesses representations of medication prescribed for personal use, in this case b/tsDMARDs, and the BMQ-General which assesses beliefs about medicines in general. The BMQ-Specific consists of ten items assessing beliefs about the necessity of prescribed medication (Specific-Necessity, five items) and concerns about prescribed medication based on beliefs about the danger of dependence and long-term toxicity and the disruptive effects of medication (Specific-Concerns, five items). The BMQ-General comprises two subscales assessing beliefs that medicines are harmful, addictive or poisons which should not be taken continuously (General-Harms, five items) and that medicines are overused by doctors (General-Overuse, three items). Responses are graded on a scale of 1 (strongly disagree) to 5 (strongly agree). Internal consistency for each of the four BMQ subscales was checked using Cronbach’s alpha, which for this data was 0.72 for BMQ Specific Concerns, 0.94 for BMQ Specific Necessity subscales, 0.76 for BMQ General Harms and 0.81 for BMQ General Overuse. The BMQ was designed for use in chronic disease [12] and has previously been used in trials of people with RA [13] and IBD [14].
4. Single Item Literacy Screener (SILS): this single-item tool’s purpose is to screen for limited reading ability, a factor of relevance to health literacy. The item asks how often respondents need assistance in reading health information materials (on a scale from 1—always to 5—never). Scores of 1 or 2 are considered to indicate some difficulty with reading printed health-related material. It has been used and validated in people with chronic diseases such as diabetes mellitus [15] and has previously been compared with other tools assessing literacy in people with IA [16].

### Statistical analysis

Data analyses and tabulations were performed in Stata v16 (StataCorp LLC, Texas, USA).

BMQ-harms, BMQ-overuse, biologic-specific necessity and biologic-specific concern scores were calculated by summing participant scores within each of the respective domains, with at most two missing responses allowed, in order to calculate a mean score (ranging from 1 to 5), as previously described [17]. Higher scores indicate that medications are perceived to be more harmful, overused, necessary or concerning respectively.

The combinations of b/tsDMARD data sources (Additional file 1: Q1.4) utilised by study respondents were visualised in an upset chart, using the R library UpsetR [18, 19].

Results were summarised as proportions of medians with interquartile (IQR) range, as appropriate.

Groups were compared statistically using a bootstrapped (5000 replicates) test for continuous variables, chi-square for unordered categorical variables, and a non-parametric trend test for ordered categorical variables.

The number of information sources utilised for each participant was analysed by multivariable Poisson regression, with covariates of patient group, sex, age more than 60, b/tsDMARD BMQ specific subscales and SILS scores. Cragg’s Hurdle model was used for a more complete analysis of the information sources selected and the favourability information obtained for covariates patient group, age, b/tsDMARD BMQ specific subscales and SILS scores.

## Results

Thousand two hundred and ninety six people responded to the survey in the time period that it was made available and there were 458 exclusions for the following reasons: no relevant diagnosis (n = 86), never heard of biologics (n = 155), no survey question responses (n = 148) and no relevant medication history (n = 69).

**Fig. 1:**
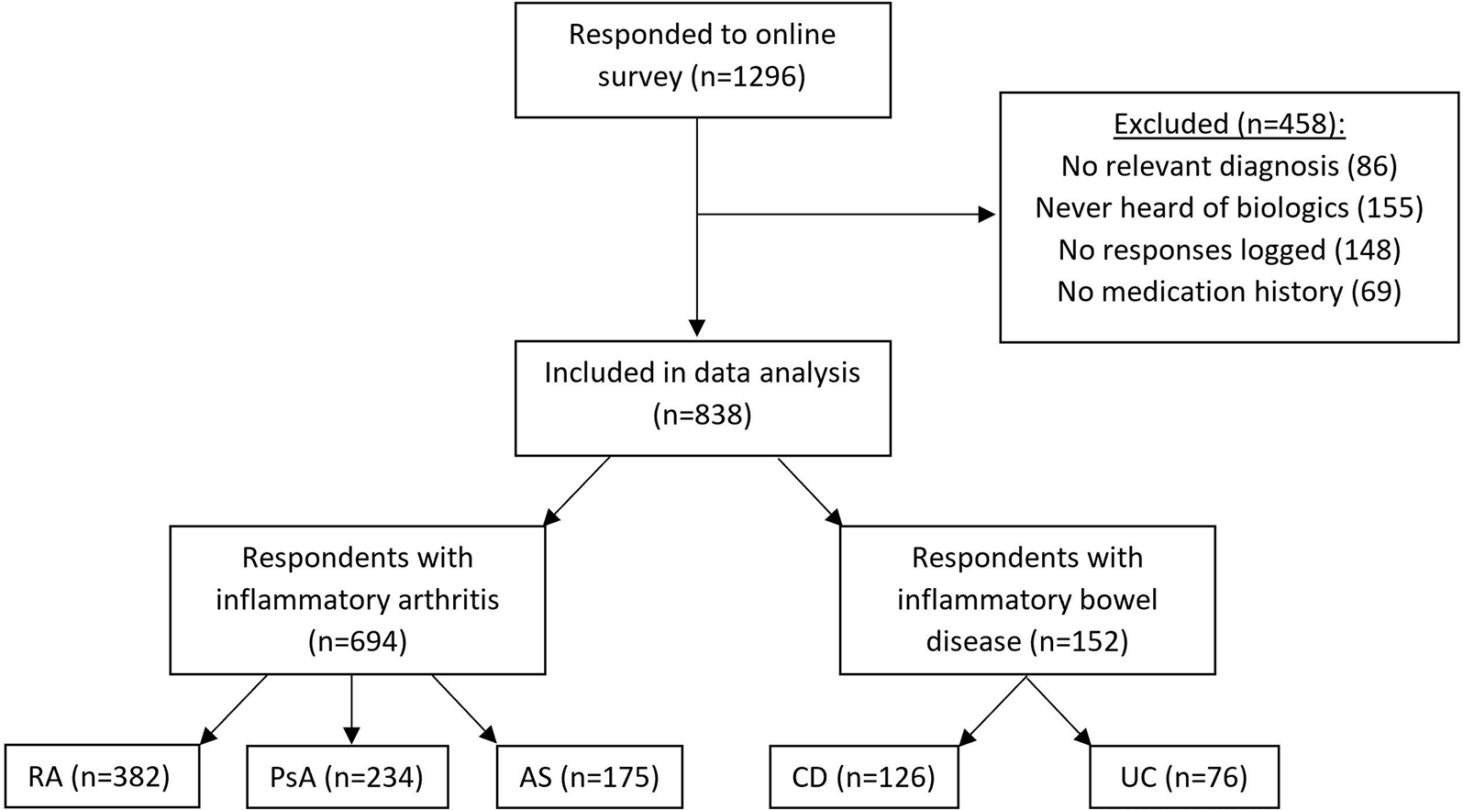
Responses to online survey and self-nominated diagnoses, noting that 164 respondents nominated more than one diagnosis

Data of 838 respondents were included, 694 (82.8%) with IA (382 (46%) with RA, 234 (28%) with psoriatic arthritis, 175 (21%) with ankylosing spondylitis) and 152 with IBD (126 (15%) with Crohn’s Disease and 76 (9%) with ulcerative colitis). 164 respondents (19.6%) nominated more than one diagnosis. Figure 1 summarises the survey responses by diagnosis.

### Participant characteristics

Demographic data of respondents by disease grouping (IA or IBD) and their reading ability and beliefs about medicines are shown in Table 1. The median age was 52 years (IQR 41, 62), 87% were female and the median disease duration was 10 years (IQR 5, 20). Gender proportions were similar across disease groups while respondents with IBD were significantly younger than those with IA (median difference 13 years, 95% CI 9.5, 16.5) with longer disease duration (median difference 3 years, 95% CI 0.5, 5.5).

**Table 1:** Study participant demographics, biologic use, SILS and Beliefs about Medicines Questionnaire results

Comparator	All	Rheumatology	Gastroenterology	*p*-value
N	838	694	144^a^	
Age in years, median (IQR)	52 (41,62)	54 (43, 63)	41 (33, 53)	0.001
Female: n (%)	724 (87)	604 (87)	120 (83)	0.38
English main language: n (%)	824 (99)	682 (99)	142 (99)	0.55
State/territory: n (%)				
New South Wales	222 (27)	184 (27)	38 (27)	0.004
Victoria	179 (21)	137 (20)	42 (29)	
Western Australia	147 (18)	127 (18)	20 (14)	
Queensland	144 (17)	129 (19)	15 (10)	
South Australia	71 (8)	58 (8)	13 (9)	
ACT	49 (6)	41 (6)	8 (6)	
Tasmania	18 (3)	18 (3)	5 (4)	
Northern territory	2 (0.2)	0	2 (1)	
Region: n (%)				
Metropolitan capital city	462(55)	377 (54)	85 (59)	0.20
Regional centre	282 (34)	233 (34)	49 (34)	
Rural/remote area	93 (11)	83 (12)	10 (7)	
Disease duration in years, median (IQR)	10 (5, 20)	9 (4, 20)	12 (6, 20)	0.017
Current b/ts DMARD use: n (%)	658 (79%)	541 (78)	117 (82)	0.35
Need help reading health-related materials (SILS)^b^, n (%)				
Always	4 (0.5)	4 (0.6)	0	0.77
Often	5 (0.6)	4 (0.6)	1 (0.8)	
Sometimes	47 (6)	36 (6)	11 (8)	
Rarely	128 (17)	107 (17)	21 (16)	
Never	582 (76)	482 (76)	100 (75)	
Beliefs about medicines (BMQ): 1–5 scale, higher score meaning more agreement, median (IQR)				
General medicine: overuse	2.7 (2, 3.3)	2.7 (2.0, 3.3)	2.7 (2.0, 3.3)	1.00
General medicine: harms	2 (1.6, 2.4)	2.0 (1.6, 2.4)	2.0 (1.6, 2.4)	1.00
bDMARD specific: necessity	4.2 (3.6, 4.8)	4.2 (3.6, 4.8)	4.0 (3.6, 4.8)	0.03
bDMARD specific: concerns	2.8 (2.2, 3.4)	2.8 (2.2, 3.2)	3.0 (2.2, 3.6)	0.14

^a^8/694 (1%) of patients reporting IA also had IBD diagnosis

^b^Single Item Literacy Screener

Most respondents were from New South Wales and Victoria. About half of the respondents were from regional and remote areas (375/837, 45%). Almost all spoke English as their main language (824/834, 99%) and 710/766 (93%) reported either never or only rarely requiring assistance in interpreting medical instructions or health-related information.

BMQ specific necessity and concern scores for biologics were similar between the IA and IBD disease groups (Table 1), with a small, but practically insignificant, difference between the biologic specific necessity subscales. Overall, there was a strong belief in the importance of biologics (BMQ b/tsDMARD Specific Necessity score 4.2 (IQR 3.6, 4.8), with a moderate level of concern (BMQ b/tsDMARD Specific Concerns score 2.8 (IQR 2.2, 3.4). Similarly, there were moderate concerns about harms and overuse of medicine in general.

### Perceptions of biologics

**Fig. 2:**
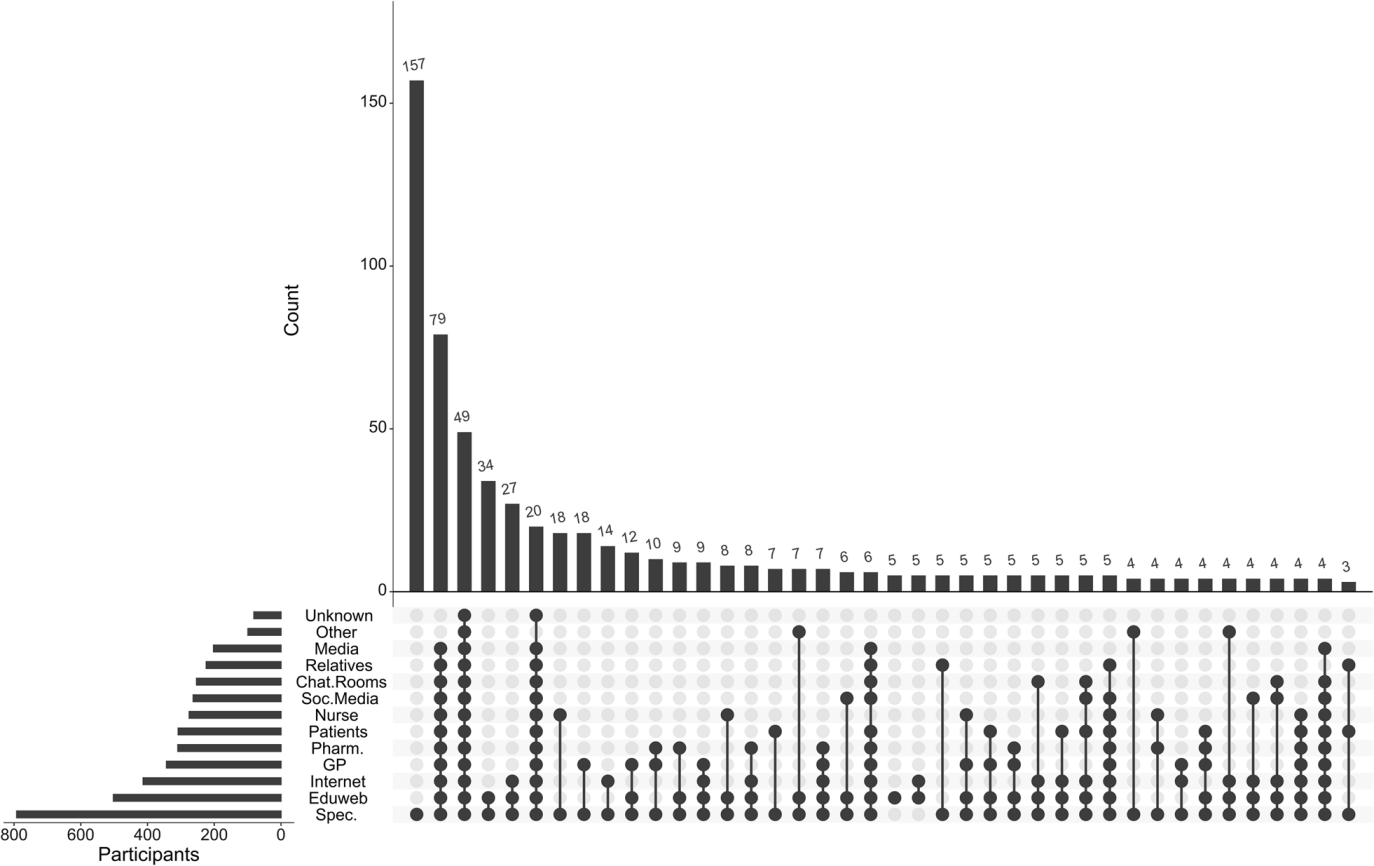
Sources of information about biologics as nominated by survey participants

Figure 2 shows the sources of information about biologics that respondents sought. Almost all (95%) nominated specialists as an information resource, and almost a fifth (18.7%) reported that specialists were their sole source. For the remainder, the median number of information sources was 4 (IQR 2–7). When analysed by a multivariable Poisson regression, the number of utilised information sources was greater for participants with higher BMQ b/tsDMARD Specific Concerns scores (*p* < 0.001), lower reading ability (SILS) scores (*p* = 0.014) and aged over 60 (*p* < 0.001), but not associated with gender (*p* = 0.92), rheumatological or gastroenterological condition (*p* = 0.99) or BMQ b/tsDMARD Specific necessity scores (*p* = 0.21).

**Fig. 3:**
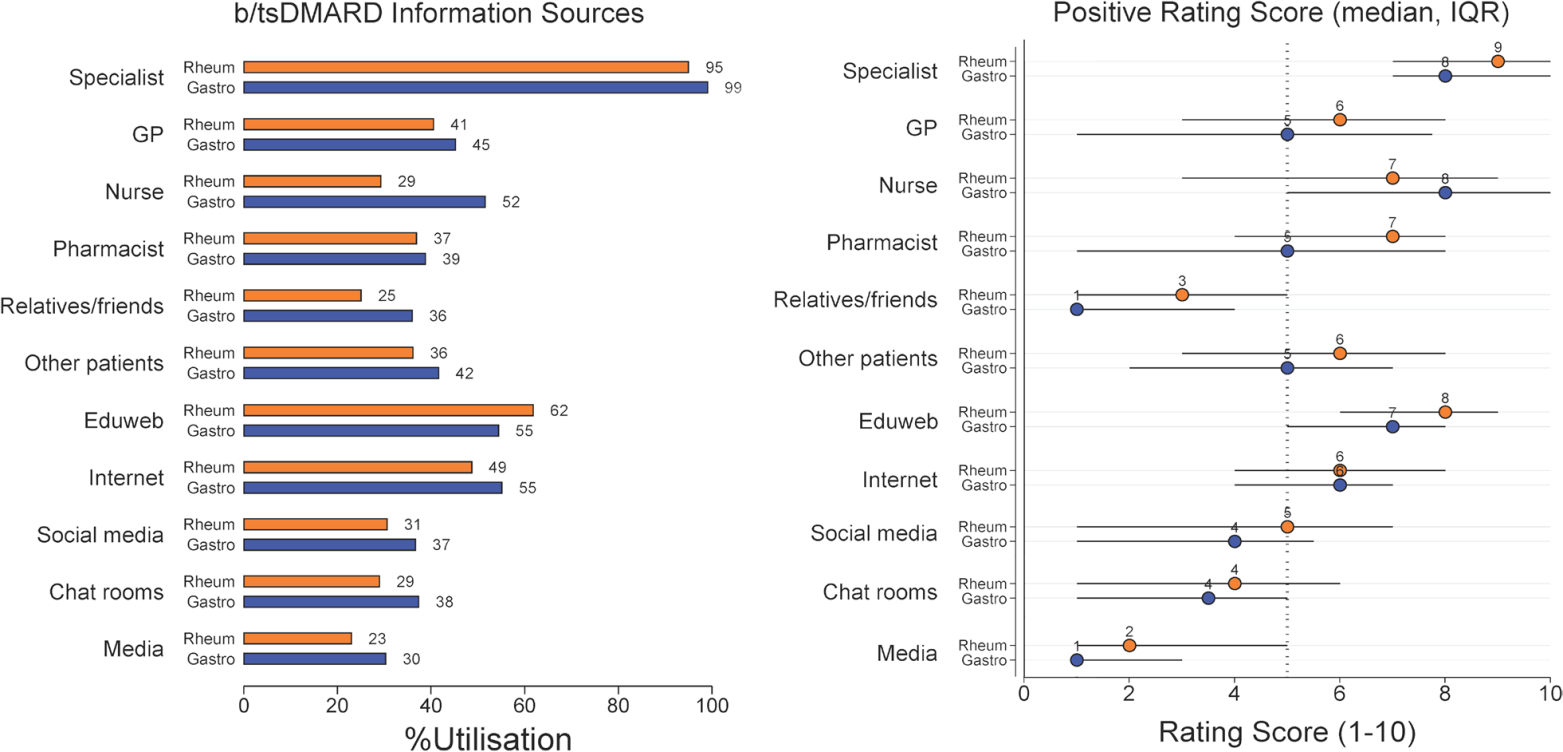
Information sources about biologic/targeted synthetic DMARDs and how positive different sources were about these medications for rheumatology and gastroenterology patients

Sources of information about biologics were generally similar when comparing IA and IBD respondents, with none reaching a statistically significant difference except for specialist nursing, which was more frequently accessed by those with IBD (IBD—73/141, 52% vs IA—202/685, 29%, *p* = 0.012) (Fig. 3).

Figure 3 also displays how favourable each source of information was considered to be by IA and IBD respondents. In general, health care practitioners (specialists, GPs and nurses) were considered to provide generally positive information about biologics (median 9, IQR 7–10) whereas social media (median 4, IQR 1–6), chat rooms (median 4, IQR 1–6) and relatives/friends (median 2, IQR 1–5) were considered to provide less favourable information.

Analysis of these information sources and favourability scores for covariates patient group, age, BMQ specific subscales and SILS scores are reported in Additional file 2: Table S1. Irrespective of diagnosis, respondents requiring help reading health-related materials were more likely to consult their GP (coefficient − 0.15, *p* = 0.04) whereas those with a higher BMQ biologic-specific concern score were more likely to use less reliable resources such as social media (coefficient 0.13, *p* = 0.04). Overall, the internet was frequently used as a source of information (educational websites, 502/838, 59.9%; other internet websites 413/838 49.2%; social media 263/838, 31.4%; chat rooms 253/838, 30.1%).

### Perceptions of biosimilars

**Fig. 4:**
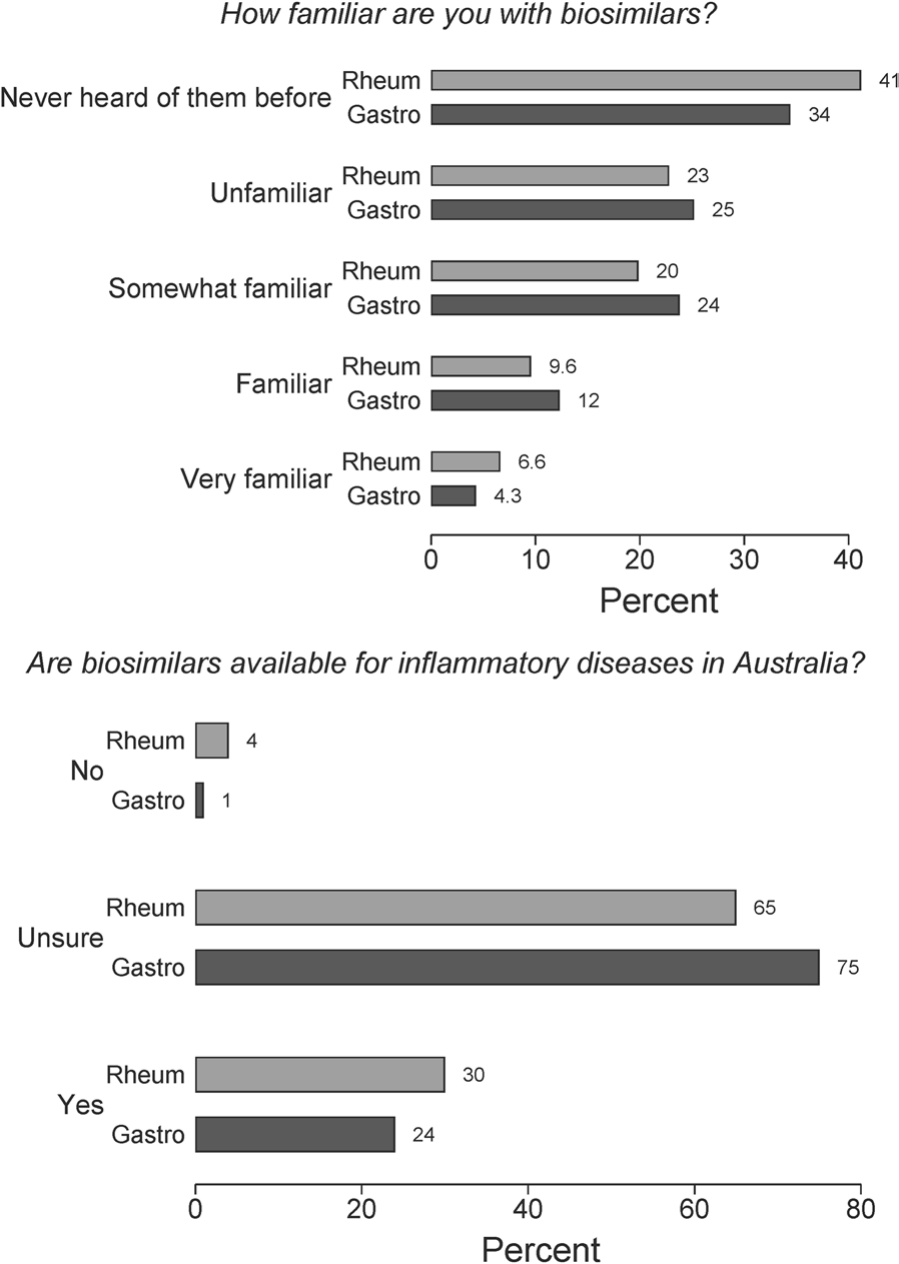
Familiarity with biosimilars and understanding of their availability in Australia

As shown in Fig. 4, about one-third of respondents who answered the questions on biosimilars had never heard of them before (280/787, 35.6%) and two-thirds of the remainder (336/502, 66.9%) were unsure if they were available in Australia. Only 23 respondents (4.6%) indicated that they were currently taking a biosimilar.

Both IA and IBD respondents indicated that specialist recommendation (352/495, 71.1%) and proven safety/efficacy from clinical trials (286/495, 57.8%) would most influence them to switch from a bio-originator to a biosimilar. Lesser reported influences were personal (123/495, 24.8%) and government (65/495, 13.1%) costs.

## Discussion

Biologic therapies increasingly play a central role in the treatment of autoimmune disease in patients who are refractory to conventional DMARDs. While evidence of their efficacy and tolerability is compelling, patient perspectives on these therapies are less well understood. In this study, we examined patient perspectives on biologics but also their sources of information about these therapies.

Our findings highlight the importance of specialists in providing information about biologics to patients but also the recognition that people frequently obtain information from multiple sources, some of which (such as relatives, friends or social media) are not favourable towards these medications. Importantly, respondents in this survey who were more concerned about biologics were more likely to use social media for information though it is difficult to ascertain the directionality of this relationship.

Respondents with IA and IBD were generally similar, with the younger age and longer disease duration of IBD patients explained by the typical peak age of onset of IBD being in the 20s with 10–20% of cases diagnosed in childhood [20] compared with RA which is most common in middle age [21].

Specialist nurses are well recognised as key parts of patients’ healthcare teams [22, 23], and our study highlights the disparity that exists between rheumatology and gastroenterology in this area, reflecting that in Australia, there are only approximately 50 rheumatology nurses to support 1.7 million people with IA [24] compared with 120 IBD nurses for 100,000 people with IBD [25].

The very low proportion of respondents in this survey who were currently using biosimilars is surprising and calls into question whether patients are aware of being on biosimilars or bio-originators and if they are being appropriately informed about switching, as we were not able to confirm the actual medication the patients were using.

Performing a questionnaire such as this one on a national scale, with two comparable populations accessing similar medications, is unique. Our results are similar to prior investigation of the attitudes of patients with rheumatoid arthritis towards methotrexate, where it was reported that specialists were the most frequently consulted and most favourable towards the drug whereas internet resources were more variable in their perspectives, with social media and chat rooms being generally negative [17].

The role of social media as an information source is becoming increasingly realised. It provides an extensive resource of information, discussion and shared experiences which can be empowering for patients and encourage them to discuss their condition and treatment options with healthcare providers [26]. However, the perils of inconsistent, unreliable or misinterpreted information have also been particularly realised in the current pandemic situation [27]. Though our results suggest that social media is generally negative in its perspective on biologics, previous social media mining suggests that public sentiment towards b/tsDMARDs is still more positive than in regard to conventional synthetic DMARDs [28].

At the time that this survey was conducted, biosimilars for infliximab, etanercept, adalimumab and rituximab were registered with the Therapeutic Goods Administration (TGA) in Australia. Though the proportion of respondents in the present study who had heard of biosimilars (64.4%) may seem low, it is higher than in surveys published in the last five years involving participants with IA [29] as well as IBD [30] where typically, less than 50% of respondents are aware of biosimilars. It also highlights the rapid increase in awareness of biosimilars amongst patients in a short span of time when compared with an Australian cross-sectional study of patients with RA in a public tertiary-referral hospital rheumatology clinic in 2017, prior to the introduction of the etanercept biosimilar to the Australian PBS, where only 6% of patients had pre-existing knowledge of biosimilars [31].

It has previously been noted that patients on biosimilars had greater confidence in their efficacy, trusted their doctor’s decision to use biosimilars and perceived that their use reduced costs to the health care system; in contrast, those on bio-originators thought that the cost of treatment should not influence prescribing [32]. This may explain why the present study’s respondents, almost all of whom reported that they were on bio-originators, were hesitant towards and placed low importance on the financial benefits of biosimilars.

The implications of this current study are significant to the clinical practice and service delivery of rheumatology and gastroenterology. A consistent message in our results which has also been found in previous surveys of patient attitudes to biologics overseas [11] is the importance of specialists in information provision, reinforcing the ongoing need to be consistent, up-to-date and accurate regarding biologics and biosimilars, while simultaneously aware that there may be intersecting or contrasting messages obtained from resources outside the clinical interaction. Similarly, patient factors such as reading ability and general concern about medications need to be considered so that information is patient-centred and personalised.

Increased training and implementation of rheumatology specialist nurses is also a key area of need in Australia. As demonstrated by respondents with IBD, information from a specialist nurse is valuable but for people with IA, limited significantly by access and availability.

A key strength of the present study was gathering information from around Australia in people with IA and IBD, to assess if despite having quite different diseases, they have similar experiences regarding biologic medications. Another strength was the integration of contextual factors (demographics, beliefs about medications and reading ability) to allow a more granular appreciation of possible determinants of information resource selection.

There are some salient limitations to mention. Comparisons between respondents with IA and IBD were limited by the inequality in the sample sizes of each group and consequently, results are skewed towards the IA group. There is also an inherent selection bias in an online survey written in English and distributed through consumer groups which may not reflect real life clinic patient populations. Collection of data on socioeconomic status and formal education levels may have allowed further analysis of data when combined with the SILS. The survey was self-reported and relied on respondents’ understandings of their diagnoses as well as medications, which is not always accurate. Finally, this data was collected in 2020 and awareness of biologics, particularly biosimilars, may well have increased since then, along with more positive attitudes towards them with more frequent prescription and acceptance by physicians and patients.

## Conclusion

Respondents with IA and IBD in this survey had generally similar positive attitudes towards biologics, but were less familiar with biosimilars. They consulted a number of information sources similarly but differed in reliance on specialist nurses which likely reflects the greater number of gastroenterology nurses compared with rheumatology nurses proportional to their patient populations in Australia. Both groups relied heavily on specialist recommendations regarding their treatment decisions, emphasising that as clinicians, our interactions and advice must continually strive to be of high quality, tailored to patients’ health literacy and targeted to their healthcare beliefs.

## Supplementary Information

**Additional file 1: Questionnaire: **Attitudes of People with Inflammatory Disease to Biologic therapy version 7 (final version).

**Additional file 2: Table S1.** Cragg’s hurdle model for utilised b/tsDMARD information sources and favourability rating of the information received.

## Data Availability

The datasets used and/or analysed during the current study are available from the corresponding author on reasonable request.

## Abbreviations

AS: Ankylosing spondylitis
BMQ: Beliefs about medicines questionnaire
b/tsDMARD: Biologic and targeted synthetic disease modifying anti-rheumatic drugs
DMARD: Disease modifying anti-rheumatic drug
IA: Inflammatory arthritis
IBD: Inflammatory bowel disease
PBS: Pharmaceutical benefits scheme
PsA: Psoriatic arthritis
RA: Rheumatoid arthritis
SILS: Single item literacy screener
TGA: Therapeutic goods administration
