# Supplementary material for: Perceptions towards biologic and biosimilar therapy of patients with rheumatic and gastroenterological conditions
Source: BMC Rheumatol. 2022 Dec 23;6:79. doi: 10.1186/s41927-022-00309-4 (PMC9783393; doi:10.1186/s41927-022-00309-4)
Supplement: Supplementary file 1 — Additional file 1: Questionnaire: Attitudes of People with Inflammatory Disease to Biologic therapy version 7 (final version). [file 41927_2022_309_MOESM1_ESM.pdf]

Attitudes of People with Inflammatory Disease to Biologic therapy

The survey is designed to investigate the knowledge and beliefs of people with inflammatory conditions regarding biologic disease modifyingmedicines (including biosimilars), and their sources of information. Inflammatory conditions include rheumatoid arthritis, psoriatic arthritis, ankylosing spondylitis, psoriasis, Crohn's Disease and ulcerative colitis. The information provided will inform positive campaigns to increase awareness and understanding of these medicines and how to take them.

Please note that completing this survey will imply consent for us to use the information you provide for the purposes of our research. However, you are able to withdraw at any time during the survey prior to completion and your responses will not be saved or included in the study. All information collected in this study will be de-identified. Your participation in this study shall not affect any other right to compensation you may have under common law.

This project will be carried out according to the National Statement on Ethical Conduct in Human Research (2007) incorporating all updates. This statement has been developed to protect the interests of people who agree to participate in human research studies.The study has been approved by the Central Adelaide Local Health Network Human Research Ethics Committee. If you wish to speak to someone not directly involved in the study about your rights as a volunteer, or about the conduct of the study, you may also contact the CALHN HREC Chairperson, on 7117 2229 or 8222 6841."

The CALHN reference number is 12423.

We expect this survey will take you about 15 minutes to complete. The closing date for the survey is XXXX.

Demographics

Year of birth

Gender
M
F
Other

In what state do you live
(list)

Do you live in a
- Metropolitan capital city
- Regional centre
- Rural/remote area

Main language spoken at home
English
Other - please specify

Do you have any of the following conditions?
Rheumatoid arthritis
Psoriatic arthritis
Ankylosing spondylitis
Crohn’s disease
Ulcerative colitis
Chronic plaque psoriasis
None of the above. (if this is selected, finish survey)

Disease duration

Year symptoms of your condition first appeared
Year your condition was initially diagnosed

 © Robert Horne 1999

Biologic medicines

What is a biologic medicine?
Biological disease modifying drugs are a group of newer medicines that are used to treat some inflammatory diseases. These medicines block natural processes which contribute to the disease process in inflammatory diseases. Biologic medicines are complex and are produced using living cells.

Types:
Biologic medicines used for inflammatory diseases in Australia are listed below:

- Abatacept (Orencia)
- Adalimumab (Humira)
- Certolizumab (Cimzia)
- Etanercept (Enbrel, Brenzys)
- Golimumab (Simponi)
- Guselkumab (Tremfya)*
- Infliximab (Remicade)
- Ixekizumab (Taltz)*
- Risankizumab (Skyrizi)*
- Rituximab (Mabthera, Riximyo)
- Secukinumab (Cosentyx)
- Tildrakizumab (Ilumya)*
- Tocilizumab (Actemra)
- Ustekinumab (Stelara)
- Vedolizumab (Entyvio)*

In addition, there is another group of medications called targeted synthetic disease modifying drugs (tsDMDs) that have recently come onto the market.

- Baricitinib (Olumiant)
- Tofacitinib (Xeljanz)

How familiar are you with biologic medicines?

- Very familiar
- Familiar
- Somewhat familiar
- Unfamiliar
- Never heard of them before (go to Section B)

Are you currently on a biologic or tsDMD?

- Yes (skip next question)

If yes, which one?

- No (go to next question)

I am not on a biologic or tsDMD because:

- I used to take a biologic, but it was stopped. If so, please advise why it was stopped (eg didn't work/side effects etc)
- I was offered a biologic, but decided not to take it. If so, please advise why you decided not to take it.
- I was not offered a biologic by my doctor.

 © Robert Horne 1999

Where did you get your information about biologic or tsDMD medicines from? (Please tick any applicable one)	Tick box	Was this advice positive (Please Rate from 1-10, 10 being the most positive and 1 very negative)
Specialist (eg rheumatologist, gastroenterologist, dermatologist)		0
General Practitioner		0
Specialist Nurse		0
Pharmacist		
Relatives or friends		0
Other people with my condition/s		0
Internet Educational Website (e.g Australian Rheumatology Association, Arthritis Australia)		0
Other internet websites (Google, Wikipedia, etc..)		0
Social media (Facebook, Twitter, Instagram)		
Online forums or chat rooms		0
Media (newspapers, magazine, television, radio)		0
Other (please specify)		
Don’t know/can’t say		

How satisfied are you with the information you have received about your biologic or tsDMARD medication? (5 pt Likert scale)

If the effectiveness of all biologic or tsDMARD medications were equal, the most important factor influencing my choice of biologic or tsDMARD would be

a. Cost of the medication to me
b. How it is given (injection under the skin that I give myself/intravenous drip/ tablet)
c. Ease of access to the medication
d. Recommendation of my specialist
e. Recommendation of my pharmacist
f. Recommendation of my specialist nurse
g. Recommendation by close friends/ family
h. Information obtained online or via media
i. Other (please specify)

Comments

YOUR VIEWS ABOUT
Biologics
(BMQ SPECIFIC)

- We would like to ask you about your personal views about biologic or tsDMD prescribed for your inflammatory condition
- These are statements other people have made about their medicines.
- Please show how much you agree or disagree with them by ticking the appropriate box.

There are no right or wrong answers.
We are interested in your personal views

	Views about **BIOLOGIC PRESCRIBED FOR YOU:**	**Scale Stucture**				
	SPECIFIC NECESSITY	**Strongly Agree**	**Agree**	**Uncertain**	**Disagree**	**Strongly Disagree**
	My health, at present, depends on my biologic or tsDMD	5	4	3	2	1
	My life would be impossible without my biologic or tsDMD	5	4	3	2	1
	Without my biologic or tsDMD I would be very ill	5	4	3	2	1
	My health in the future will depend on my biologic or tsDMD	5	4	3	2	1
	My biologic or tsDMD protects me from becoming worse	5	4	3	2	1
	SPECIFIC CONCERN	**Strongly Agree**	**Agree**	**Uncertain**	**Disagree**	**Strongly Disagree**
	Having to take a biologic or tsDMD worries me	5	4	3	2	1
	My biologic or tsDMD is a mystery to me	5	4	3	2	1
	My biologic or tsDMD disrupts my life	5	4	3	2	1
	I sometimes worry about becoming too dependent on my biologic or tsDMD	5	4	3	2	1
	I sometimes worry about the long term effects of my biologic or tsDMD	5	4	3	2	1

All items scored : 5= strongly agree, 4= agree, 3= uncertain, 2= disagree, 1= strongly disagree

Comments

	**Views about BIOLOGIC OR tsDMD PRESCRIBED FOR YOU:**	**Scale Stucture**				
	COMPARED WITH other DMDS*	**Strongly Agree**	**Agree**	**Uncertain**	**Disagree**	**Strongly Disagree**
	Biologic or tsDMD medications have less side effects than other DMARDs	5	4	3	2	1
	Biologic or tsDMD medications are stronger than other DMDs	5	4	3	2	1
	Biologic or tsDMD medications suppress the immune system more than other DMARDs	5	4	3	2	1
	Biologic or tsDMD medications are more toxic than other DMARDs	5	4	3	2	1
	Biologic or tsDMD medications make me more likely to get infections than other DMDs	5	4	3	2	1

* The most common other, non-biologic DMDs are methotrexate, sulfasalazine, hydroxychloroquine, and leflunomide. Less frequently used medications include gold, azathioprine, and cyclosporine.

All items scored : 5= strongly agree, 4= agree, 3= uncertain, 2= disagree, 1= strongly disagree

Evidence shows that some people with well-controlled disease can safely reduce the dose of their biologic or tsDMD or how frequently they take it, without any worsening of their condition. If your treating specialist suggested it, would you be willing to try taking your biologic or tsDMD less frequently, or at a reduced dose?

Yes
No
Maybe

YOUR VIEWS ABOUT
Biosimilars

Section A. Your knowledge about biosimilars

How familiar are you with biosimilar medicines?

- Very familiar
- Familiar
- Somewhat familiar
- Unfamiliar
- Never heard of them before (go to Section B)

1. What does the term 'biosimilar medicine' (biosimilar) mean?
   a. Not sure
   b. A biologic medicine that is identical to an existing (originator) biologic but made by a different company
   c. A generic version of an existing (originator) biologic which may be less expensive
   d. A close copy of an existing (originator) biologic which has been shown to have no significant difference to the originator biologic.

2. Are biosimilars available for treatment of inflammatory diseases in Australia?
   a. Yes
   b. No
   c. Not sure

	How much do you agree with the statements below about biosimilars?	Scale Stucture				
		Strongly Agree	Agree	Uncertain	Disagree	Strongly Disagree
	Biosimilars are as safe as biologics	5	4	3	2	1
	Biosimilars are as effective as biologics	5	4	3	2	1
	I would be willing to take a biosimilar if my physician suggests it	5	4	3	2	1
	I would require more information about biosimilars before deciding whether to take them.<br>If so, what information would you need?	5	4	3	2	1

1. I am currently prescribed a biosimilar by my rheumatologist
   a. Yes (which one)
   b. No
   c. Not sure

Where did you get your information about biosimilars from (Please tick any applicable one)	Tick box	Was this advice positive (Please Rate from 1-10, 10 being the most positive and 1 very negative)
Specialist (eg rheumatologist, gastroenterologist, dermatologist)		0 100
General Practitioner		0 100
Specialist Nurse		0 100
Pharmacist		
Relatives or friends		0 100
Other people with my condition/s		0 100
Internet Educational Website (e.g Australian Rheumatology Association, Arthritis Australia)		0 100
Other internet websites (Google, Wikipedia, etc..)		0 100
Social media (Facebook, Twitter, Instagram)		0 100
Online forums or chat rooms		0 100
Media (newspapers, magazine, television, radio)		0 100
Other (please specify)		0 100
Don't know/can't say		0 100

Section B - What is a Biosimilar?

A biosimilar medicine is a copy of a biologic medicine, a bit like a generic medicine. For many years, generic medicines, which are identical copies of the original medicine, have been available as less expensive alternatives to traditional brands.

Unlike generic medicines, however, it is not possible to make an exact copy of a of biologic medicines because of the manufacturing process. Biologic medicines are complex and are produced using living cells so they are naturally variable.

However, it is possible to make a highly similar copy of a biologic product and this is referred to as a "biosimilar". The term "biosimilar" refers to a biologic product which has been developed and tested to ensure that there is no significant difference between the "biosimilar" and the original biologic.

1. Do you use generic brands of other medicines (such as paracetamol, aspirin, ibuprofen, atorvastatin)?
    a. Yes
    b. No
    c. Not sure

2. What do you feel are the main differences between biologics (originators) and biosimilars? (You may choose more than one answer)
    a. Quality
    b. Effectiveness
    c. Safety
    d. Price

e. None of the above

3. How confident are you or would you be in taking a biosimilar medicine?
   a. Totally confident
   b. Very confident
   c. Neutral
   d. Little confidence
   e. Not confident at all

4. When the price of the biologic (originator) is the same as the biosimilar, which one would you prefer to be treated with?
   a. Biologic (originator)
   b. Biosimilar
   c. Whichever my rheumatologist thought would be best for my condition
   d. Not sure

5. When the price to the government of the biologic (originator) is more than the biosimilar, which one would you prefer to be treated with?
   a. Biologic (originator)
   b. Biosimilar
   c. Whichever my rheumatologist thought would be best for my condition
   d. Depend how much the price difference is
   e. Not sure

6. If you were/ are on treatment with a biologic (originator) when would you consider switching to a biosimilar? (you may choose multiple answers)
   a. Never
   b. If my rheumatologist recommended it
   c. When clinical trials had proven that the safety and effectiveness of the biosimilar was equal to the biologic (originator) for my disease
   d. When the cost to me was less than the biologic (originator)
   e. When the cost to the government was less than the biologic (originator)
   f. If the biosimilar was more convenient for me

YOUR VIEWS ABOUT
MEDICINES IN GENERAL (BMQ GENERAL)

- These are statements that other people have made about medicines in general.
- Please show how much you agree or disagree with them by ticking the appropriate box.

	Views about MEDICINES IN GENERAL	Strongly Agree	Agree	Uncertain	Disagree	Strongly Disagree
	Doctors use too many medicines	5	4	3	2	1
	People who take medicines should stop their treatment for a while every now and again	5	4	3	2	1
	Most medicines are addictive	5	4	3	2	1
	Natural remedies are safer than medicines	5	4	3	2	1
	Medicines do more harm than good	5	4	3	2	1
	All medicines are poisons	5	4	3	2	1
	Doctors place too much trust on medicines	5	4	3	2	1
	If doctors had more time with patients they would prescribe fewer medicines	5	4	3	2	1

All items scored : 5= strongly agree, 4= agree, 3= uncertain, 2= disagree, 1= strongly disagree

Comments

 © Robert Horne 1999

We are interested in the other medications that you may be on.

**ARE YOU ON methotrexate?**
I am currently taking methotrexate<br>(if yes dose? And how is it administered? Oral or injection)
I used to take methotrexate but it was stopped (because it didn't work/side effects/I didn't want to take it anymore _______________________)
I was offered methotrexate but I decided not to take it, because________

**ARE YOU ON leflunomide (also called Arava, Arabloc or Ataris))?**
I am currently taking leflunomide<br>(if yes dose?)
I used to take leflunomide but it was stopped (because it didn't work/side effects/I didn't want to take it anymore _______________________)
I was offered leflunomide but I decided not to take it, because________

**ARE YOU ON prednisolone?**
I am currently taking prednisolone<br>(if yes daily dose?)<br>(if yes, take intermittently vs take everyday?)<br>(If yes, what year were you started on prednisolone?)
I used to take prednisolone but it was stopped because …………..
I was offered prednisolone but I decided not to take it, because________

**ARE YOU ON opioids (eg codeine, oxycodone, tramadol, fentanyl)**
I am currently taking opioids<br>(if yes daily dose?)<br>(if yes, take intermittently vs take everyday?)<br>(If yes, what year were you started on prednisolone?)
I used to take prednisolone but it was stopped because …………..
I was offered prednisolone but I decided not to take it, because________

"How often do you need to have someone help you when you read instructions, pamphlets, or other written material from your doctor or pharmacy?" Never/Rarely/Sometimes/Often/Always
