# Supplementary material for: Perceptions towards biologic and biosimilar therapy of patients with rheumatic and gastroenterological conditions
Source: BMC Rheumatol. 2022 Dec 23;6:79. doi: 10.1186/s41927-022-00309-4 (PMC9783393; doi:10.1186/s41927-022-00309-4)
Supplement: Supplementary file 2 — Additional file 2: Table S1. Cragg’s hurdle model for utilised b/tsDMARD information sources and favourability rating of the information received. [file 41927_2022_309_MOESM2_ESM.docx]

Khoo et al.

**Supplementary Table S1:** Cragg’s hurdle model for utilised b/tsDMARD information sources and favourability rating of the information received.

The analysis has two components: a selection component for whether an information source was utilised or not, and a score component for how favourable the views were when the information source was utilised. A positive coefficient in the selection component indicates the covariate was associated with a higher likelihood of that specific information source being utilised. Similarly, a positive coefficient in the score component indicates the covariate was associated with a more favourable rating for that specific information source.

Models for each information source, and combined using seemingly unrelated regression (suest command in Stata)

| Covariate | Coef | Std.Err | p_val | Covariate | Coef | Std.Err | p_val |
| --- | --- | --- | --- | --- | --- | --- | --- |
| **Specialist: selection** |  |  |  | **Specialist: score** |  |  |  |
| Gastroenterology | 0.410 | 0.425 | 0.34 | Gastroenterology | 0.370 | 0.212 | 0.081 |
| Age | -0.021 | 0.009 | 0.024 | Age | 0.005 | 0.006 | 0.38 |
| BMQ specific necessity | 0.477 | 0.103 | < 0.001 | BMQ specific necessity | 0.629 | 0.107 | < 0.001 |
| BMQ specific concerns | 0.330 | 0.171 | 0.053 | BMQ specific concerns | -0.935 | 0.099 | < 0.001 |
| Health literacy | 0.002 | 0.183 | 0.99 | Health literacy | 0.467 | 0.169 | 0.006 |
| **GP: selection** |  |  |  | **GP: score** |  |  |  |
| Gastroenterology | -0.134 | 0.137 | 0.33 | Gastroenterology | -0.849 | 0.633 | 0.18 |
| Age | -0.011 | 0.004 | 0.005 | Age | 0.066 | 0.017 | < 0.001 |
| BMQ specific necessity | 0.048 | 0.058 | 0.41 | BMQ specific necessity | 0.657 | 0.254 | 0.010 |
| BMQ specific concerns | 0.208 | 0.063 | < 0.001 | BMQ specific concerns | -0.524 | 0.262 | 0.045 |
| Health literacy | -0.151 | 0.072 | 0.037 | Health literacy | 0.068 | 0.300 | 0.82 |
| **Specialist Nurse: selection** | |  |  | **Specialist Nurse: score** |  |  |  |
| Gastroenterology | 0.337 | 0.134 | 0.012 | Gastroenterology | 1.479 | 0.531 | 0.005 |
| Age | -0.016 | 0.004 | < 0.001 | Age | -0.010 | 0.021 | 0.63 |
| BMQ specific necessity | 0.036 | 0.058 | 0.53 | BMQ specific necessity | 0.364 | 0.291 | 0.21 |
| BMQ specific concerns | 0.037 | 0.064 | 0.57 | BMQ specific concerns | -0.795 | 0.299 | 0.008 |
| Health literacy | -0.095 | 0.074 | 0.20 | Health literacy | 0.274 | 0.364 | 0.45 |
| **Pharmacist: selection** |  |  |  | **Pharmacist: score** |  |  |  |
| Gastroenterology | -0.236 | 0.139 | 0.090 | Gastroenterology | -1.079 | 0.761 | 0.16 |
| Age | -0.016 | 0.004 | < 0.001 | Age | 0.027 | 0.018 | 0.14 |
| BMQ specific necessity | 0.042 | 0.058 | 0.47 | BMQ specific necessity | 0.334 | 0.286 | 0.24 |
| BMQ specific concerns | 0.039 | 0.063 | 0.53 | BMQ specific concerns | -0.683 | 0.272 | 0.012 |
| Health literacy | -0.176 | 0.072 | 0.015 | Health literacy | 0.053 | 0.301 | 0.86 |
| **Relatives: selection** |  |  |  | **Relatives: score** |  |  |  |
| Gastroenterology | 0.094 | 0.142 | 0.51 | Gastroenterology | -4.382 | 10.791 | 0.69 |
| Age | -0.014 | 0.004 | < 0.001 | Age | 0.052 | 0.139 | 0.71 |
| BMQ specific necessity | -0.071 | 0.060 | 0.23 | BMQ specific necessity | -0.487 | 1.573 | 0.76 |
| BMQ specific concerns | 0.174 | 0.067 | 0.009 | BMQ specific concerns | 0.008 | 1.247 | 1.00 |
| Health literacy | -0.109 | 0.075 | 0.15 | Health literacy | -1.249 | 2.935 | 0.67 |
| **Other patients: selection** | |  |  | **Other patients: score** |  |  |  |
| Gastroenterology | -0.096 | 0.137 | 0.48 | Gastroenterology | -0.522 | 0.617 | 0.40 |
| Age | -0.017 | 0.004 | < 0.001 | Age | 0.010 | 0.018 | 0.57 |
| BMQ specific necessity | -0.019 | 0.058 | 0.75 | BMQ specific necessity | 0.120 | 0.266 | 0.65 |
| BMQ specific concerns | 0.103 | 0.063 | 0.10 | BMQ specific concerns | -0.247 | 0.286 | 0.39 |
| Health literacy | -0.054 | 0.074 | 0.47 | Health literacy | 0.472 | 0.335 | 0.16 |
| **Educational websites: selection** | |  |  | **Educational websites: score** | |  |  |
| Gastroenterology | -0.334 | 0.133 | 0.012 | Gastroenterology | -0.698 | 0.411 | 0.089 |
| Age | -0.011 | 0.004 | 0.004 | Age | 0.010 | 0.010 | 0.32 |
| BMQ specific necessity | 0.078 | 0.057 | 0.17 | BMQ specific necessity | 0.397 | 0.156 | 0.011 |
| BMQ specific concerns | 0.098 | 0.062 | 0.11 | BMQ specific concerns | -0.571 | 0.158 | < 0.001 |
| Health literacy | -0.025 | 0.075 | 0.74 | Health literacy | 0.255 | 0.212 | 0.23 |
| **Other internet: selection** | |  |  | **Other internet: score** |  |  |  |
| Gastroenterology | -0.085 | 0.134 | 0.53 | Gastroenterology | -0.252 | 0.389 | 0.52 |
| Age | -0.016 | 0.004 | < 0.001 | Age | -0.005 | 0.012 | 0.67 |
| BMQ specific necessity | 0.012 | 0.056 | 0.84 | BMQ specific necessity | 0.367 | 0.182 | 0.044 |
| BMQ specific concerns | 0.119 | 0.061 | 0.050 | BMQ specific concerns | -0.301 | 0.190 | 0.11 |
| Health literacy | -0.032 | 0.074 | 0.66 | Health literacy | 0.338 | 0.203 | 0.096 |
| **Social media: selection** | |  |  | **Social media: score** |  |  |  |
| Gastroenterology | -0.101 | 0.142 | 0.47 | Gastroenterology | -1.359 | 1.167 | 0.24 |
| Age | -0.017 | 0.004 | < 0.001 | Age | -0.048 | 0.040 | 0.22 |
| BMQ specific necessity | 0.018 | 0.060 | 0.77 | BMQ specific necessity | -0.175 | 0.450 | 0.70 |
| BMQ specific concerns | 0.131 | 0.064 | 0.042 | BMQ specific concerns | -0.142 | 0.484 | 0.77 |
| Health literacy | -0.070 | 0.076 | 0.36 | Health literacy | 0.344 | 0.562 | 0.54 |
| **Chat rooms: selection** |  |  |  | **Chat rooms: score** |  |  |  |
| Gastroenterology | -0.069 | 0.140 | 0.62 | Gastroenterology | -1.557 | 0.851 | 0.067 |
| Age | -0.019 | 0.004 | < 0.001 | Age | -0.040 | 0.028 | 0.16 |
| BMQ specific necessity | 0.041 | 0.061 | 0.50 | BMQ specific necessity | -0.201 | 0.373 | 0.59 |
| BMQ specific concerns | 0.111 | 0.065 | 0.090 | BMQ specific concerns | -0.233 | 0.391 | 0.55 |
| Health literacy | -0.090 | 0.075 | 0.23 | Health literacy | 0.081 | 0.449 | 0.86 |
| **Other media: selection** | |  |  | **Other media: score** |  |  |  |
| Gastroenterology | -0.023 | 0.146 | 0.88 | Gastroenterology | -1.315 | 1.169 | 0.26 |
| Age | -0.010 | 0.004 | 0.011 | Age | 0.043 | 0.037 | 0.24 |
| BMQ specific necessity | -0.037 | 0.063 | 0.55 | BMQ specific necessity | -0.055 | 0.355 | 0.88 |
| BMQ specific concerns | 0.169 | 0.069 | 0.014 | BMQ specific concerns | 0.527 | 0.482 | 0.27 |
| Health literacy | -0.072 | 0.078 | 0.36 | Health literacy | 0.133 | 0.584 | 0.82 |

- The key differences between respondents with inflammatory arthritis (IA) and inflammatory bowel disease (IBD) are for the specialist nurse and educational websites information sources for biological therapies. Respondents with IBD are more likely to consult/report information from specialist nurses, and additionally report more favourable views. 202/685 (29%) of respondents with IA reported views from a specialist nurse compared to 73/141 (52%) of respondents with IBD. Conversely, respondents with IA tended to consult/report information from educational websites and additionally report more favourable views. 425/485 (62%) of respondents with IA reported views from educational websites compared to 77/141 (55%) of respondents with IBD.
- There are a number of other inferences for other covariates from this analysis:
- Younger respondents are more likely to consult/report information from multiple different sources
- Respondents with poorer health literacy are more likely to consult/report information from GPs and pharmacists
- Respondents with higher biologic BMQ specific concerns are more likely to consult/report less reliable information sources such as other internet, social media and other media
- Respondents with higher biologic BMQ specific necessity and lower concerns scores report more favourable views of biologics obtained from specialists, GPs and educational websites.
